# Retention in Care and Connection to Care among HIV-Infected Patients on Antiretroviral Therapy in Africa: Estimation via a Sampling-Based Approach

**DOI:** 10.1371/journal.pone.0021797

**Published:** 2011-07-26

**Authors:** Elvin H. Geng, David V. Glidden, Mwebesa Bosco Bwana, Nicolas Musinguzi, Nneka Emenyonu, Winnie Muyindike, Katerina A. Christopoulos, Torsten B. Neilands, Constantin T. Yiannoutsos, Steven G. Deeks, David R. Bangsberg, Jeffrey N. Martin

**Affiliations:** 1 Division of HIV/AIDS at San Francisco General Hospital, Department of Medicine, University of California San Francisco, San Francisco, California, United States of America; 2 Department of Epidemiology and Biostatistics, University of California San Francisco, San Francisco, California, United States of America; 3 Department of Medicine, Mbarara University of Science and Technology, Mbarara District, Republic of Uganda; 4 Department of Medicine, Center for AIDS Prevention Studies, University of California San Francisco, San Francisco, California, United States of America; 5 Division of Biostatistics, Department of Medicine, Indiana University, Indianapolis, Indiana, United States of America; 6 Massachusetts General Hospital, Harvard Medical School, Boston, Massachusetts, United States of America; 7 East Africa International Epidemiologic Databases to Evaluate AIDS (IeDEA) Consortium, Eldoret, Kenya; University of Cape Town, South Africa

## Abstract

**Introduction:**

Current estimates of retention among HIV-infected patients on antiretroviral therapy (ART) in Africa consider patients who are lost to follow-up (LTF) as well as those who die shortly after their last clinic visit to be no longer in care and to represent limitations in access to care. Yet many lost patients may have “silently” transferred and deaths shortly after the last clinic visit more likely represent limitations in clinical care rather than access to care after initial linkage.

**Methods:**

We evaluated HIV-infected adults initiating ART from 1/1/2004 to 9/30/2007 at a clinic in rural Uganda. A representative sample of lost patients was tracked in the community to obtain updated information about care at other ART sites. Updated outcomes were incorporated with probability weights to obtain “corrected” estimates of retention for the entire clinic population. We used the competing risks approach to estimate “connection to care”—the percentage of patients accessing care over time (including those who died while in care).

**Results:**

Among 3,628 patients, 829 became lost, 128 were tracked and in 111, updated information was obtained. Of 111, 79 (71%) were alive and 35/48 (73%) of patients interviewed in person were in care and on ART. Patient retention for the clinic population assuming lost patients were not in care was 82.3%, 68.9%, and 60.1% at 1, 2 and 3 years. Incorporating updated care information from the sample of lost patients increased estimates of patient retention to 85.8% to 90.9%, 78.9% to 86.2% and 75.8% to 84.7% at the same time points.

**Conclusions:**

Accounting for “silent transfers” and early deaths increased estimates of patient retention and connection to care substantially. Deaths soon after the last clinic visit (potentially reflecting limitations in clinical effectiveness) and disconnection from care among patient who were alive each accounted for approximately half of failures of retention.

## Introduction

Assessing the effectiveness of the global effort to provide antiretroviral therapy (ART) for HIV-infected patients in resource limited settings must quantify both the number of patients enrolling in care as well as the number of patients who continue to access care over time [1]. Despite the importance of understanding engagement with ART services, however, efforts to quantify this experience have been hampered by both conceptual and practical barriers. First, existing estimates of retention — which have concluded that as few as 60% of patients who start ART in Africa are retained after two years — have been conducted from the perspective of individual clinics and have assumed that patients who are lost to follow-up (i.e., who have unknown outcomes) are no longer engaged in care [2], [3], [4], . In the setting of rapid scale up and decentralization of ART services, this assumption may not be true. Patients who fail to return to the clinics where they started ART may in fact have desirable outcomes (i.e., transfers to newer and closer ART delivery sites) as well as poor outcomes (i.e., deaths or disengagement from care) [7]. Second, many deaths occur very shortly after the last visit to clinic. Existing approaches combine these patients with patients who are alive but who disengage from care and consider both as “not retained.” Deaths shortly after the last visit, however, are more likely due to limitations in the effectiveness of medical care (because of limited diagnostic or therapeutic options) and therefore represent a different problem than disengagement from the clinic after enrollment (potentially due to transportation, stigma, and other socio-structural factors). Alternative analytic approaches that are able to distinguish these early deaths after a clinic visit from disengagement from care should be explored.

To address these barriers, first we use a sampling-based approach to identify outcomes among those patients who are lost to follow-up from their original ART clinic. By tracking and obtaining updated information (e.g., vital status, continued receipt of HIV care) from a small but representative sample of patients who become lost to follow-up, an accurate estimate of retention in care for all patients in the clinic population who initiate ART can be derived. Of note, while we have previously reported the fraction of patients among those lost to follow-up who “silently” transferred care to a different facility (where the denominator is the numerically small sample of lost patients who were tracked and interviewed) [7], in this analysis we take the next step by using the sampling-based approach to estimate retention in care for the entire, larger clinic population. Second, we apply a competing risk approach estimate the percentage of patients accessing care and which distinguishes deaths soon after the clinic visit from disengagement from care. This estimate, which we term “connection to care,” is a proposed metric of the ability of patients to attend clinic.

## Methods

### Ethics Statement

The study was approved by the institutional review board of University of California, San Francisco, and the Mbarara University of Science and Technology. The ethics approvals for this study allow for the analysis of de-identified clinic data collected in the course of routine care and monitoring activities, therefore specific written or verbal consent was waived.

### Patients

The patient population has been previously described [7]. In brief, we evaluated all HIV-infected adults attending the Immune Suppression Syndrome (ISS) Clinic in Mbarara, Uganda, who initiated ART between January 1, 2004 and September 30, 2007. The ISS Clinic opened on January 21, 1998, but the number of patients was limited to less than a hundred until scale-up occurred in 2004 and 2005, when the clinic enrolled up to 350 patients and initiated 200 patients a month on ART. By October 1, 2007, the total clinic enrollment had reached 12,915, and the cumulative ART patients were 4,986. Services at the clinic are supported by the Uganda Ministry of Health, two implementing partners of the President's Emergency Plan for AIDS Relief and a private foundation (Family Treatment Fund). The clinic serves Mbarara Municipality, which has a population of 82,000, and the rural districts of Mbarara, Bushenyi, Ntungamo and Isingiro. These districts cover a radius of approximately 60 km around Mbarara Municipality with a total population of 1.7 million persons in 2006 [8].

Patients were followed from ART initiation to either death or administrative database closure on September 30, 2007. In July 2006, the ISS Clinic began a program in which a tracker went into the community to determine the outcomes of an unselected and consecutive sample of patients who had become lost to follow-up. Each month the clinic's electronic medical record system generated a list of patients who were lost to follow-up, defined as not being seen at the clinic for at least 6 months. The tracker sought to locate and directly speak with the missing patients or close informants (e.g., family members, neighbors, or friends) in person and in the community. A proxy was interviewed only if the patient him or herself could not be interviewed because he/she was living or working in a distant location or was not found at home after repeated visits there. Given that time was limited relative to the large number of lost patients, the tracker was unable to search for all lost patients in all months. Therefore, effectively only a sample of patients was sought.

### Measurements

Demographic and clinical characteristics were obtained from the ISS Clinic's electronic medical record system. Among patients who were lost to follow-up and found in the community by the tracker, the clinic also administered a short structured questionnaire to determine current access to HIV care despite absence from the ISS Clinic. Specifically, this questionnaire asked *“Have you seen a doctor or nurse for the care or treatment of HIV within the last three months?”* and *“Have you been taking antiretroviral medications for the treatment of HIV in the last 30 days?”* If the patient answered affirmatively to either question, the site of HIV care was solicited. Patients were considered to be “in HIV care” if (a) they answered affirmatively to both questions and (b) they provided a legitimate site for both the clinic visits and the source of ART.

### Statistical Analyses

We sought to estimate both “retention in care”, defined as the fraction of all patients starting ART who continue to be alive and access HIV care, irrespective of clinic site, and a new parameter termed “connection to care.” This new parameter is conceptually defined as the percentage of ART initiators who either continue to be alive and access care or who died while in care (i.e., shortly after the last clinic visit). The motivation for estimating connection to care is the recognition that deaths often occur despite recent contact with an HIV clinic (i.e., despite being connected to care). Many of the major causes of AIDS related deaths in Africa (such as smear-negative tuberculosis, visceral Kaposi's sarcoma, etc.) can be difficult to diagnose without sophisticated testing capabilities [9], [10], carry high mortality even when treated [11], [12], and are therefore beyond the capacity of many new ART clinics to avert. Conceptually, retention in care combines patients who (a) die early after their last visit to clinic with (b) those patients who cease to access care and who may subsequently die later. These two outcomes, however, represent very different processes: early deaths are due to a limitation of clinical care in most cases whereas patients who cease coming to clinic (and who may die later because of this lapse) do so because of stigma, transportation costs, or other socio-structural barriers. Connection in care, therefore, attempts to capture the fraction of patients who kept a reasonable level of contact with ART services – even if they died despite this contact – and therefore provides another metric of access to ART services in Africa. When estimating both retention in care and connection to care, we conducted a naïve analysis, which used only data on patient outcomes that were routinely available to the ISS Clinic through passive means, and a corrected analysis in which we incorporated updated data on vital status and current HIV care obtained from the sample of lost patients who were tracked in the community.

For all time to event analyses, ART initiation was time zero. In the naïve estimation of “retention in care,” the Kaplan Meier technique was used to estimate time to failure of retention (defined as 3 months after expected return visit or death) [13]. In the naïve estimation of “connection to care,” the cumulative incidence approach [14], [15], [16] using competing risks was used to estimate the occurrence of becoming “disconnected from care,” again defined as being 3 months late for a return visit. However, in this estimate, deaths that occurred within 3 months of the expected return to clinic were treated as a competing risk. In other words, this analysis considers disconnection from care as the event of interest and deaths that occur shortly after the last clinic as a competing risk because these early death alter the probability of the event of interest. All other patients were censored at the date of their last clinic visit.

In the corrected analyses, the same general analytic approaches were used. Lost patients who were tracked and found to be in HIV care at a clinic different than the ISS Clinic were censored on the date of interview by the tracker. Lost patients who were found alive but not in HIV care at any site were considered to have the event of “disconnection from care” 3 months after their expected return date at the ISS Clinic. If a tracked patient could not be found in person but was deemed to be alive by report of an informant (spouse, child, parent, neighbor, etc.), we did not ask that informant whether the patient was still in HIV care because this could inadvertently disclose the HIV status of the patient. Therefore, we conducted a sensitivity analysis under a “pessimistic” assumption that all patients who were alive but not directly interviewed were no longer in HIV care and then under an “optimistic” assumption that all patients who were reported to be alive actually remained in HIV care. All corrected estimates were derived using sampling-based probability weights [17], [18], where outcomes in the sample of successfully tracked patients represent outcomes in all other patients lost to follow-up. Confidence intervals (CI) in corrected analyses were derived through bootstrapping. Finally, we conducted a logistic regression of factors associated with retention in care at a new site among the sample of patients who were lost to follow-up, tracked and interviewed in person. Socio-demographic factors associated with retention at a significance level of <0.1 were included in a multivariable model.

## Results

Characteristics of this patient population have been previously reported but are represented for completeness (Table 1) [7]. In brief, a total of 3628 HIV-infected adults newly initiating ART were evaluated. The median age was 35 years (interquartile range (IQR) 30 to 42), and 61% were women. The median CD4+ T cell count prior to ART in 1954 patients in whom it was available was 95 cells/mm^3^ (IQR 36 to 172). The number of new patients starting therapy was 522 in 2004, 1465 in 2005, 850 in 2006 and 796 between January 1 and September 30, 2007.

**Table 1:** Patient characteristics.

	All Patients (Group A)	Patients Lost to Follow-up (Group B)	Patients Lost to Follow-up and Tracked (Group C)	Patients Tracked with Vital Status Ascertained (Group D)	Lost Patients Without Vital Status Ascertainment (Group E)
Total No.	3628	829	128	111	718
Female (%)	61	58	59	59	58
Age (years), median (IQR)*	35 (30–42)	36 (30–42)	35 (29–42)	35 (29–42)	36 (31–42)
Pre-therapy CD4 count (cells/µl), median (IQR)†	95 (36–172)	72 (19–150)	90 (20–187)	75 (20–191)	72 (19–144)
WHO stage 3 or 4 (%)‡	71.9	79.5	77.3	76.6	79.9
ART start date, median (IQR)	15-Nov-05 (30-Mar-05 to 13-Nov-06)	10-Jun-05 (10-Jan-05 to 28-Oct-05)	20-Aug-05 (06-Apr-05 to 30-Oct-05)	30-Aug-05 (08-Apr-05 to 14-Nov-05)	29-Apr-05 (04-Jan-05 to 26-Oct-05)
District of residence					
Bushenyi	574 (15.82)	185 (22.32)	26 (20.31)	22 (19.82)	163 (22.7)
Isingiro	428 (11.80)	49 (5.91)	9 (7.03)	8 (7.21)	41 (5.71)
Mbarara	1,790 (49.34)	358 (43.18)	70 (54.69)	61 (54.95)	297 (41.36)
Ntungamo	287 (7.91)	70 (8.44)	5 (3.91)	5 (4.5)	65 (9.05)
Other	549 (15.13)	167(20.14)	18 (14.06)	15 (13.51)	152 (21.17)
Last CD4 value before loss to follow- up (days), median (IQR)	NA	77 (20–163)	92 (20–192)	91 (20–192)	76 (20–161)
Length on ART before loss to follow-up (days), median (IQR)	NA	217 (15–365)	242 (36–377)	243 (29–380)	213 (14–357)
Length of time between last clinic visit and tracking (months), median (IQR)	N/A	N/A	11.8 (9.5–14.3)	12.0 (9.4–14.3)	10.8 (9.6–12.7)

*Age was missing in 30 patients.

† The last CD4 value in the 6 months before starting therapy was missing in 1954 patients.

‡ WHO stage at the time of initiation of ART was missing in 273 patients.

### Tracking a Sample of Losses to Follow-up

Over a maximum of 3.75 years of observation, 829 patients became lost to follow-up as defined by 6 months of absence from the ISS Clinic. A total of 128 of the 829 patients were sought after in the community by a tracker, and in 111 of 128 cases (87%) updated information on vital status was obtained. Patients who were lost and subsequently had outcomes ascertained by tracking (n = 111) were relatively similar to all patients who were lost to follow-up in age (median 35 vs. 36 years), gender (59% vs. 58% women), median pre-therapy CD4+ T cell count (75 vs. 72 cells/µl), ART start date (August 30, 2005 vs. June 10^th^, 2005), residence in Mbarara District (55% vs. 43%), last CD4 count before lost to follow-up (91 vs. 77 cells/µl), length of time on ART before loss in follow-up (243 vs. 217 days) and other patient characteristics (Table 1). Among 128 lost to follow-up patients who were tracked, the length of time between last visit and tracking was similar for 111 patients who were successfully tracked (i.e., who had at least updated vital status ascertained) compared to the 28 who did not (12.8 months vs. 10.8 months). Among the 111 patients whose outcomes were updated through tracking in the community, 79/111 (71%) were found to be alive. Of these 79, 48 were directly interviewed, and 31 had information provided by an informant.

### Retention in Care and Connection to Care

Among the 48 patients whom had been deemed lost but who were found alive and directly interviewed upon tracking in the community, 35 (73%) indicated they were in HIV care as evidenced by having (a) seen a provider for HIV care in the past 3 months, (b) taken ART in the 30 days, and (c) provided legitimate sites for HIV care. The most common care site reported was The AIDS Support Organization (TASO), where 10 patients reported receiving care. The rest reported recently receiving care at 20 various health centers in the region. Of the remaining 13 patients directly interviewed, 8 reported neither seeing an HIV provider in the last 3 months nor taking ART in the last 30 days and 5 reported having made a visit to an HIV provider but that they had not taken ART in the previous 30 days.

**Figure 1:**
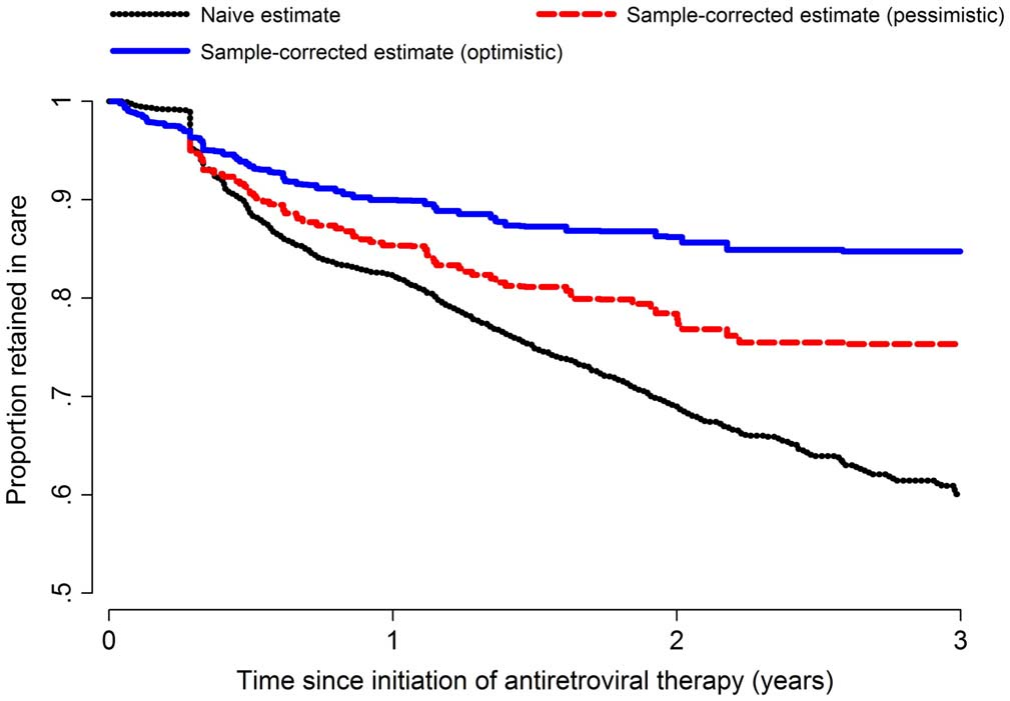
Naïve and corrected plots of “retention in care.” Retention in care is defined as the fraction of patients who remain alive and in HIV care. The naïve estimate assumes that all patients who are lost to follow-up from the ISS clinic are no longer retained in care. The corrected estimates of retention in care are based on outcomes ascertained from a sample of patients who were lost to follow-up from the ISS Clinic, sought in the community and in whom updated information about vital status and HIV care was obtained. If a tracked patient was found to be alive by report of an informant, we did not ask that informant whether the patient was still in HIV care because this could inadvertently violate the privacy of the patient. Therefore, we conducted a sensitivity analysis under two assumptions. The “pessimistic” corrected estimate is based on the assumption that all patients who were alive but not directly interviewed in person were no longer in HIV care. The “optimistic” corrected estimate was based on the assumption that all patients who were reported to be alive but not directly interviewed in person remained in HIV care.

An estimate of retention for the entire clinic population under the “naïve” assumption (that patients lost to follow-up from the ISS clinic were no longer in HIV care), retention in care was 82.3% (95% CI: 80.9–83.7), 68.9% (95% CI: 67.1–70.8), and 60.1% (95% CI: 57.3–62.7) at 1, 2 and 3 years after ART initiation. The estimate of retention for the clinic population after incorporating updated care and ART status from the sample of lost patients, but under the pessimistic assumptions of the sensitivity analysis (that patients who were reported alive by an informant were not in HIV care), was 85.8% (95% CI: 82.7–88.9), 78.9% (95% CI: 75.2–82.6) and 75.8% (95% CI: 71.6–80.1). Under the optimistic assumption in the sensitivity analysis (that patients found alive but not interviewed in person were all in care), retention in care for the clinic population at the same time points was 90.9% (95% CI: 87.3–92.7), 86.2% (95% CI: 82.9–89.5) and 84.7% (95% CI: 81.0–88.5) (Figure 1 and Table 2).

**Table 2:** Naïve and corrected estimates of connection to care and retention in care with 95% confidence intervals.

	Naïve		Corrected (pessimistic)		Corrected (optimistic)	
Time since ART initiation(years)	Connection to care	Retention in care	Connection to care	Retention in care	Connection to care	Retention in care
**1**	83.8% (82.5–85.1)	82.3% (80.9–83.7)	90.9% (88.1–93.8)	85.8%(82.7–88.9)	95.1% (93.3–96.4)	90.9% (87.3–92.7)
**2**	70.8% (68.9–72.6)	68.9% (67.1–70.8)	85.8% (81.9–89.8)	78.9% (75.2–82.6)	93.2% (90.9–95.1)	86.2% (82.9–89.5)
**3**	62.0% (59.3–64.7)	60.1%(57.3–62.7)	83.6% (79.4–87.7)	75.8% (71.6–80.1)	92.6% (89.6–94.6)	84.7% (81.0–88.5)

Connection to care is defined as the fraction of ART initiators who are alive and accessing care or who died while in care. Retention in care is defined as the fraction of ART initiators who are alive and accessing HIV care.

**Figure 2:**
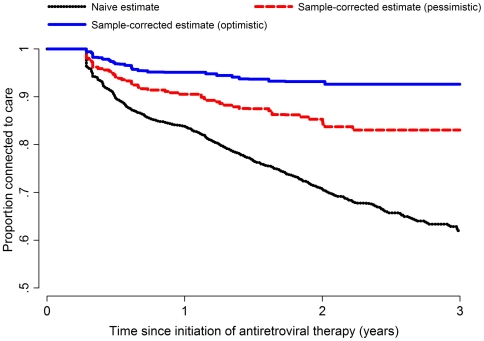
Naïve and corrected estimates of “connection to care.” Connection to care uses a competing risk approach to estimate the probability of ART initiators access care and includes patients who are alive and continuing to use the clinic as well as those died while accessing care (i.e., who died shortly after their last clinic visit).

The estimate of connection to care in the clinic population (again defined as the fraction of patients who remain in care or who died in care) – was 83.8% (95% CI: 82.5–85.1), 70.8% (95% CI: 68.9–72.6), and 62.0% (95% CI: 68.9–72.6) at 1, 2 and 3 years following ART in the naïve analysis that considered all patients lost from ISS Clinic to all be unengaged in care. In the sample-corrected estimate, under the pessimistic assumptions of the sensitivity analysis connection in care was estimated to be 90.9% (95% CI: 88.1–93.8), 85.8% (95% CI: 81.9–89.8) and 83.6% (95% CI: 79.4–87.7) at the same time points. In the sample-corrected estimates of connection to care under the optimistic assumptions of the sensitivity analysis, connection to care was estimated to be 95.1% (95% CI: 93.3–96.4), 93.2% (95% CI: 90.9–95.1) and 92.6% (95% CI: 89.6–94.6) at 1, 2 and 3 years (Figure 2).

### Factors Associated with Retention in Care among those Lost to Follow-up

Among patients who were lost to follow-up and interviewed directly, distance between the ISS Clinic and residence was the only factor significantly associated with the retention in care in unadjusted analysis: each 10 km between residence and clinic conferred a 1.30 fold rise in the odds of retention in care (95% CI, 1.04 to 1.62, p = 0.02). In a multivariable analysis adjusting for age, distance was associated with a 1.45 fold rise in the odds of retention in care (95% CI, 1.11 to 1.90, p = 0.01) and each calendar year of last visit conferred a 4.35 fold rise in the odds of retention in care (95% CI 1.23 to 15.4, p = 0.02) (Table 3).

**Table 3:** Factors associated with retention in care among a sample of patients who were lost to follow-up from the ISS Clinic (N = 48).

Factor	Unadjusted			Adjusted*		
	Odds Ratio	95% CI	p-value	Odds Ratio	95% CI	p-value
**Age (per 10 years)**	1.87	0.95–3.71	0.07	1.08	1.00–1.17	0.05
**Distance (per 10 kilometer)**	1.30	1.04–1.62	0.02	1.45	1.11–1.90	0.01
**Male sex**	0.90	0.25–3.27	0.87			
**Pre-therapy CD4+ T cell value (per 50 cells)**	1.05	0.77–1.42	0.78			
**Calendar date of last visit (per year)**	2.05	0.82–5.13	0.13	4.35	1.23–15.36	0.02

*All factors adjusted for other factors in column.

## Discussion

This study adds to the literature assessing the impact of the scale-up of ART services in Africa by providing a method for estimating retention in care that accounts for the large fraction of patients initially lost to follow-up (i.e., who have unknown outcomes). Using this method, we found that between 78.9% and 86.2% of the clinic patient population starting ART during this time were retained in care two years after ART initiation. This paints a different picture of the effectiveness of ART delivery than our naïve estimate of 68.9% or previous work which suggested at as few as 50% were retained at that time [2]. Although there is no clear threshold at which retention in care is considered adequate, these figures are disparate enough to potentially represent “failure” on the one hand and “success” on the other.

This study also proposes a metric termed “*connection to care*” that may further the epidemiologic discourse on engagement with ART services in Africa. Given a lack of sophisticated diagnostic or therapeutic capabilities, some patients (such as those with smear negative tuberculosis or malignancies) will likely die despite engagement with the package of interventions centered on ART. The metric of “retention in care” groups deaths in patients who were essentially in care at the time of death with living patients who cease to access care, many of who die after disengagement. As such, it is a measure of the total effect of ART services including the success of clinical care as well as continued access to care. The estimate of “connection to care,” in contrast, captures the fraction of patients who are in care even if they died while in care (i.e., shortly after a clinic visit) and is therefore a more precise measure of continued access to public health services in resource limited settings. Although deaths shortly after the last clinic visit may be due to a limitation of the diagnostic technologies or treatment modalities available, these deaths are less likely attributable to a problem with access to the ART care system once initial linkage was established. We found approximately half of patients who were not retained in care died before a long interval of absence from clinic (3 months) while the other half “disconnected” from care while still alive. This implies both improvement of clinical services (at the last clinic visit) and outreach (to re-engage those out of care) in equal measure are needed to optimize patient outcomes.

Although large implementing organizations and national programs are decentralizing ART services [19], few studies have documented the movement of patients from older centralized to newer and lower level ART clinics and considered the implications of this movement on estimates of retention [20]. We found that among those lost from the ISS Clinic in Mbarara, each additional calendar year of last visit conferred over a 4-fold rise in the odds of being retained in care while each 10 kilometers of distance from residence to clinic conferred almost a 50% rise in the odds of retention in care at another site. In the setting of southwestern Uganda, these associations are unsurprising: while in 2000 the ISS Clinic was the only ART delivery site in the region, by 2009 over 60 ART delivery centers had opened in southwestern Uganda. These data are consistent with the hypothesis that high loss to follow-up is in part due to “silent transfers” during the decentralization of ART services. An alternative interpretation of these data is that losses to follow-up at a particular clinic are due to a large extent to incomplete penetration of ART delivery into the community.

This study has several important limitations. First, we cannot be sure that the patients whom we deemed to be retained in care after transferring to another clinic did not also experience a break in ART with its attendant virologic and immunologic consequences [21], [22]. Research on the “safety” of transfers (whether silent or documented) is urgently required to answer this question. Second, it is possible that patients who have truly stopped ART may nonetheless report ART use due to perceived social desirability. The tracker, however, was not a medical provider and hence have had a more limited influence on reporting and all patients who reported a “silent transfer” also provided names of legitimate ART providers in the area – providing some additional support for the veracity of their reports. Third, we were unable to directly ascertain whether the 39% of patients reported to be alive by an informant were in HIV care. Sensitivity analysis, however, yielded figures that departed significantly from the naïve estimates, and hence the central inference that retention in care is underestimated remains unchanged. Finally, our sample was not formally random and we did not ascertain outcomes in 100% of the sample and so our estimates may be biased.

In summary, a sampling-based approach and competing risk analyses can provide estimates of retention in care and connection to care among HIV-infected patients on ART in Africa where resource limitations preclude ascertainment of outcomes in all patients. In estimates using both pessimistic and optimistic assumptions, we found retention in care and connection to care in a patient population of ART users in Southwestern Uganda to be higher than existing studies suggest. Among those lost, patients who lived farther from the clinic and who became lost later in calendar time were more likely to be retained in care elsewhere —an observation consistent with the presence of “silent transfers” during a period of decentralization of ART services in southwestern Uganda. Use of a sampling-based approach at “sentinel sites” or more widely within clinic based cohorts to estimate retention in care and connection to care across diverse setting can help to improve evaluation of the global effort to deliver ART.
